# Detrimental Effects of Ethanol and Its Metabolite Acetaldehyde, on First Trimester Human Placental Cell Turnover and Function

**DOI:** 10.1371/journal.pone.0087328

**Published:** 2014-02-04

**Authors:** Sylvia Lui, Rebecca L. Jones, Nathalie J. Robinson, Susan L. Greenwood, John D. Aplin, Clare L. Tower

**Affiliations:** 1 Maternal and Fetal Health Research Centre, Institute of Human Development, University of Manchester, St. Mary’s Hospital, Manchester, United Kingdom; 2 Central Manchester University Hospitals NHS Foundation Trust, Manchester Academic Health Science Centre, Manchester, United Kingdom; Institute of Zoology, Chinese Academy of Sciences, China

## Abstract

Fetal alcohol spectrum disorder (FASD) describes developmental issues from high maternal alcohol intake, which commonly results in fetal growth restriction and long term morbidity. We aimed to investigate the effect of alcohol and acetaldehyde, on the first trimester placenta, the period essential for normal fetal organogenesis. Normal invasion and establishment of the placenta during this time are essential for sustaining fetal viability to term. We hypothesise that alcohol (ethanol) and acetaldehyde have detrimental effects on cytotrophoblast invasion, turnover and placental function. Taurine is an important amino acid for neuronal and physiological development, and so, its uptake was assayed in cells and placental explants exposed to alcohol or acetaldehyde. First trimester villous explants and BeWo cells were treated with 0, 10, 20, 40 mM ethanol or 0, 10, 20, 40 µM acetaldehyde. The invasive capacity of SGHPL4, a first trimester extravillous cytotrophoblast cell line, was unaffected by ethanol or acetaldehyde (p>0.05; N = 6). The cells in-cycle were estimated using immunostaining for Ki67. Proliferating trophoblast cells treated with ethanol were decreased in both experiments (explants: 40% at 20 mM and 40 mM, p<0.05, N = 8–9) (cell line: 5% at 20 mM and 40 mM, p<0.05, N = 6). Acetaldehyde also reduced Ki67-positive cells in both experiments (explants at 40 µM p<0.05; N = 6) (cell line at 10 µM and 40 µM; p<0.05; N = 7). Only in the cell line at 20 µM acetaldehyde demonstrated increased apoptosis (p<0.05; N = 6). Alcohol inhibited taurine transport in BeWo cells at 10 mM and 40 mM (p<0.05; N = 6), and in placenta at 40 mM (p<0.05; N = 7). Acetaldehyde did not affect taurine transport in either model (P<0.05; N = 6). Interestingly, system A amino acid transport in placental explants was increased at 10 µM and 40 µM acetaldehyde exposure (p<0.05; N = 6). Our results demonstrate that exposure to both genotoxins may contribute to the pathogenesis of FASD by reducing placental growth. Alcohol also reduces the transport of taurine, which is vital for developmental neurogenesis.

## Introduction

Rates of alcohol consumption amongst women of reproductive age are steadily increasing, with almost half of all young women in the UK are reported to drink during the week and a fifth reported to binge drink [1]. Chronic high alcohol intake during pregnancy is associated with fetal alcohol spectrum disorder (FASD), which encompasses a range of developmental problems, including characteristic facial features, altered neurodevelopment, cognitive and behavioural disabilities and fetal growth restriction (FGR). It is recognised that FASD is entirely preventable through alcohol abstinence but worldwide 30%, and up to 60%, of pregnant women consume alcohol during pregnancy [2]–[5].

Diagnosis of FASD is difficult due to phenotypic variation and it is often a diagnosis of exclusion [6]. One of the most consistent features of FASD is FGR [7]. Poor placental development is a major underlying pathology; placentas from pregnancies with FGR are lower in weight, have increased apoptosis and reduced cell proliferation [8] and are characterised by a more superficial invasion of trophoblast into uterine spiral arteries [9], [10]. FGR is also associated with altered placental function, in particular reduced activity of amino acid transporters [11], [12]. Efficient nutrient transport throughout pregnancy is vital for normal fetal development, and alterations in placental-fetal exchange may result in ongoing insufficiency of nutrient supply to the fetus and suboptimal health in later life [13], [14].

Alcohol (ethanol) and its teratogenic metabolite acetaldehyde freely cross the placenta, and accumulate in fetal blood at concentrations similar to those found in maternal blood [15]–[17]. Length of fetal exposure to alcohol is entirely dependent on maternal metabolism, which varies between women [17]. Despite alcohol being the most common and widely available social drug, and its association with FGR, relatively little is understood regarding its effects on the developing placenta in human pregnancies, particularly in the earliest stages of pregnancy.

In the mouse, continuous exposure to high levels of ethanol (20%v/v) during pregnancy decreases fetal growth, affecting pup development and mortality [18]–[20]. Even at moderate levels of exposure (6%v/v) there is significant facial dysmorphia in mice [21]. A reduction in fetal weight and neonatal growth is also observed in rats [22]. Placental development is significantly altered with increased placental weight in rats following chronic high ethanol (20%v/v) liquid diet [19], [23], [24]. This increase is accompanied by trophoblast morphological irregularities and altered blood vessel development in the nutrient-exchanging labyrinth zone [19]. In sheep on a high ethanol diet, placental transport of system A-dependent α-amino isobutyric acid is reduced [25]. Significant reductions in system A transport is also seen in human term placental tissue, where the effect of alcohol is dose-dependent and towards chronic levels [26].

Acetaldehyde, a metabolite of alcohol, has well established genotoxic effects in human [27], [28] and in animal models [29]–[31]. After exposure to acetaldehyde, it is found is freely present in the placenta, amniotic fluid and fetal liver in rat and sheep, and has been shown to decrease offspring size with obvious head sparing [22], [30].

Reports of the effects of alcohol on the human placenta have mostly concentrated on term tissue which may not be adequately representative of the early stages of pregnancy, when optimal placental development is critical, and when women are more likely to consume alcohol due to unrecognised pregnancy [32]–[35]. Furthermore, much of the experimental work is toxicologically focused, with levels of alcohol equivalent to extremely high exposure.

The current study aimed to examine the effects of ethanol and its metabolite acetaldehyde on the growth and function of first trimester placenta. We hypothesized that ethanol and acetaldehyde have detrimental effects on placental development by adversely affecting cellular turnover and migration in the first trimester human placenta and cytotrophoblast. We also hypothesized a detrimental effect on the placental transport systems for amino acids important in fetal growth and development - system A and system β [36]. System β (TauT) activity is of particular interest as it transports taurine, an essential amino acid in pregnancy that is important for fetal neurodevelopment [37].

## Materials and Methods

### Tissue Collection

This study was approved by the North West Regional Ethics Committee (08/H1010/28). Written informed consent from patients was given to collect samples from elective surgical or medical terminations of pregnancy (6–11 weeks gestation).

### Tissue/Cell Culture

First trimester placental villous explants of approximately 0.5 cm^2^ were cultured in DMEM/Ham’s F12 (1∶1) media (Lonza, Slough, UK) supplemented with penicillin/streptomycin/glutamine (PSG, 0.5 mg/ml) and 5% fetal calf serum (FCS) (5% FCS for explants and 10% FCS for cell lines). All additional media reagents were purchased from Invitrogen, Paisley, UK. At each 24 hour interval, medium was replenished with the addition of 0 mM, 10 mM, 20 mM or 40 mM ethanol, or 0 µM, 10 µM, 20 µM, or 40 µM acetaldehyde, and left for 5 minutes at room temperature before transferring back into incubators at (21% O_2_ for cell lines and 6% O_2_ for first trimester explants). BeWo cells from the European collection of cell cultures (ECACC) [38], were cultured on glass coverslips for 48 h (N = 6), in duplicate for each set of treatments. First trimester explants were cultured in triplicate for 72 h on Netwell permeable supports, which were suspended in an optimal volume of media to maximise surface area to gas ratio.

Invasion assays were performed by culturing the extravillous trophoblast cell line SGHPL4, a kind gift from Professor Guy Whitley at St George’s Hospital Medical School, London, UK. The cells were culture in duplicate, on matrigel coated, 8 µm pore membrane inserts. Media used was Ham’s F10 medium (Lonza, Slough, UK), supplemented with PSG (0.5 mg/ml) and 10% fetal calf serum. Cells were exposed to ethanol or acetaldehyde as above for 24 h.

### Immunohistochemistry

After culture, first trimester explants were fixed for 24 h in 10% neutral buffered formalin (NBF) prior to paraffin embedding. 5 µm tissue sections on poly-L-lysine (Sigma, Poole, UK) coated slides were subjected to immunohistochemistry using monoclonal antibodies to detect markers of proliferation and apoptosis: Ki67 (0.6 µg/ml, Dako, Cambridge, UK) and anti-cytokeratin M30 (1 µg/ml, Roche, West Sussex, UK) respectively, as previously described [39]. Negative controls were non-immunized mouse IgG at matching concentration to the primary antibody. BeWo cells were fixed in ice cold methanol before following with the same immunohistochemical protocol. For the invasion assay, migrated SGHPL4 cells on the underside of the filter were lightly fixed in their inserts with NBF before staining with Harris’ haematoxylin. Non-migrated SGHPL4 cells on the upper surface were gently wiped away for better imaging.

### Microscopy

A Leitz Dialux 22 microscope was used in conjunction with a QI Cam Fast 1394 camera and Image-Pro Plus 6.0 imaging system (Media Cybernetics) for photography and analysis of immunostaining.

### Immunohistochemistry Quantification

Cell turnover was quantified by the ratio of positively stained cells and total number of nuclei for Ki67 (in-cycle) and M30 (cytokeratin-18 epitope) to generate proliferative and apoptotic indices respectively. In tissue samples, cytotrophoblast and stromal layers were quantified separately. Haematoxylin-positive stained SGHPL4 cells migrated through to the underside of the pore inserts were counted for the invasion index. After imaging for proliferation, apoptotic and invasion, the average of 10–15 fields of view of each tissue section or coverslip was counted for each treatment (in duplicate for each treatment). To ensure good quantitative sampling, the data points represent the average of each treatment, which were the average of the duplicates with 10–15 fields of view per duplicate. These were presented as fold change from control to account for the variability in basal proliferation and apoptosis in tissue samples. For consistency, the data from the cell models were expressed in the same format. Data are presented as fold change from control explants/cells to account for the variability in basal proliferation and apoptosis between tissue samples. Statistical analyses were performed using Wilcoxon signed-rank tests for comparison to control.

### System β and System A Activity: ^3^H taurine and^ 14^C-MeAIB Uptake

To assess system A and system β activity, paired amino acid uptake experiments were performed in either Na^+^-containing or Na^+^-free Tyrode’s buffer as previously described [40] and the Na^+^-dependent component of uptake was determined. After culture, the first trimester placental explants were tied to steel hooks (in triplicate per treatment for each uptake) and pre-equilibrated in DMEM/Tyrode’s buffer (1∶1) containing the appropriate concentration of ethanol or acetaldehyde for 30 minutes at 37°C. Transporter activity was then assessed by 30 min incubations in Tyrode’s buffer (Na^+^-containing or Na^+^-free) containing^ 14^C-methylaminoisobutyric acid (^14^C-MeAIB) (for system A, 8.5 nmol/ml; 0.019 Mbq/ml, n = 6) or ^3^H-taurine (for system β, 50 pmol/ml; 0.037 Mbq/ml, n = 7). To stop transporter activity, the fragments were vigorously washed in ice-cold Tyrode’s buffer (Na^+^-containing or Na^+^-free) and incubated in distilled water for 19 hours to release radiolabelled amino acids taken up into the fragments. Thereafter, scintillation fluid was added to the water for radioisotope counting (TRI_CARB 2100TR, Packard Biosciences). To normalize for size of explants, the triplicate fragments for Na^+^-containing, and also the Na^+^-free, uptake assays were individually dissolved in 0.3 M NaOH at 37°C overnight. The protein content for each singular uptake was assayed using the Bradford assay method. Amino acid transporter activity for each treatment was determined by the normalized average of the three explants and then subtracting amino acid uptake in Na^+^-free conditions from that in Na^+^-containing buffer to determine the Na^+^ independent activity.

Transporter activity in BeWo cells was carried out using a similar method except cells were cultured onto 35 mm dishes (in triplicate per treatment), and radiolabelled ^14^C-MeAIB or ^3^H-taurine was added directly to the dishes for 10, 20 or 30 min. After incubations, cells were washed with ice cold Tyrode’s buffer to stop uptake and 0.3 M NaOH was added to the cells for 1–2 h; these lysates were used for both scintillation counting and protein assay. Data are presented as Na^+^-dependent ^14^C-MeAIB uptake (system A activity: pmol/mg protein) or ^3^H-taurine uptake (system β activity: fmol/mg of protein). All data are shown as fold change from control, with statistical analysis using Wilcoxon-signed rank test.

## Results

### Effect of Ethanol and Acetaldehyde on Trophoblast Invasion

**Figure 1:**
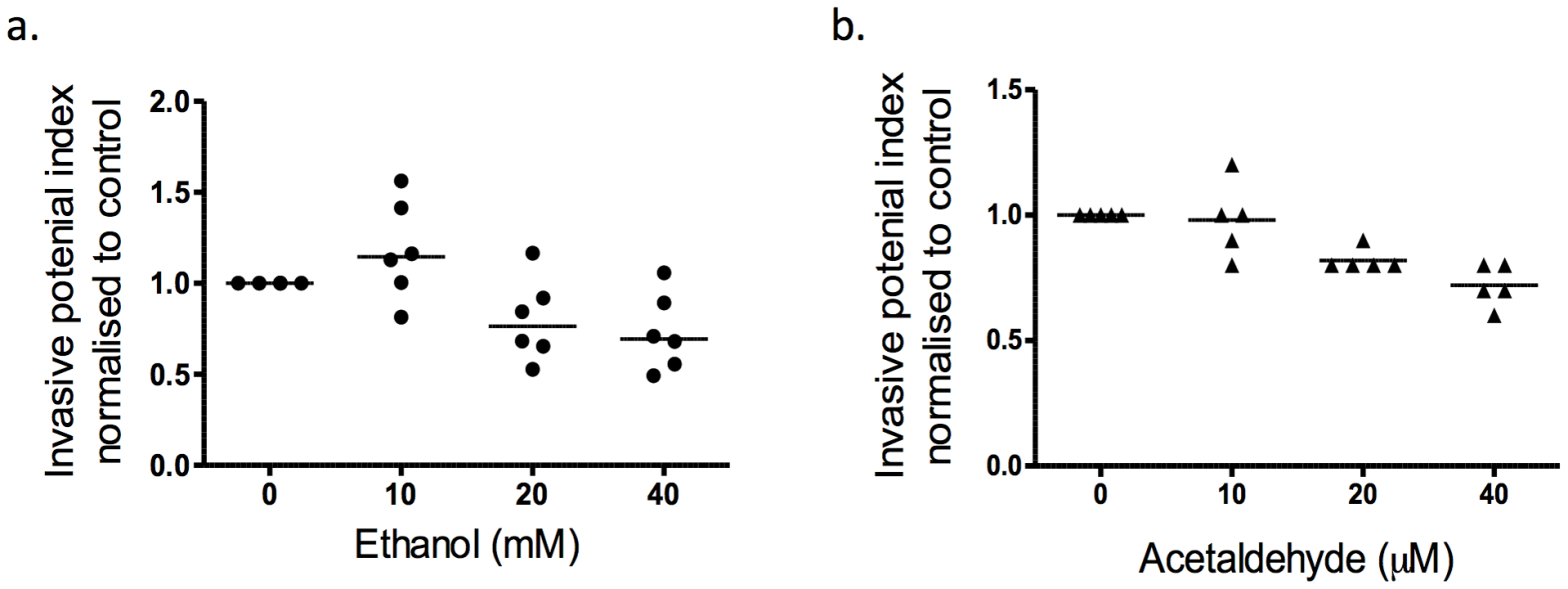
Effect of ethanol and acetaldehyde on invasive capacity of SGHPL4 extravillous cytotrophoblast cells. Invasion through a matrigel coated filter of SGHPL4 cells after treatment. (a) Invasion after exposure to ethanol. There was no effect of ethanol at any concentrations used (p>0.05; N = 6). (b) Invasion after exposure to acetaldehyde. Treatment with acetaldehyde also had no effect on the cell’s ability to invade through the matrigel-coated barrier membrane (p>0.05; N = 5). Statistical analysis with Wilcoxon signed-rank as compared to control.

SGHPL-4 cells treated with ethanol exhibited no change in invasion through the matrigel-coated membrane barrier (figure 1a; p>0.05; N = 6), but it is noteworthy that at the highest concentration, there was a strong trend for reduced in invasion with 40 mM ethanol (p = 0.06; N = 6). Exposure to acetaldehyde at 1000-fold lower concentration also had no affect (figure 1b; p>0.05; N = 5) where exposure to 20 µM and 40 µM indicated a strong trend towards inhibiting invasion (p = 0.055 and p = 0.06 respectively).

### Effect of Ethanol and Acetaldehyde on Placental Cell Turnover

**Figure 2:**
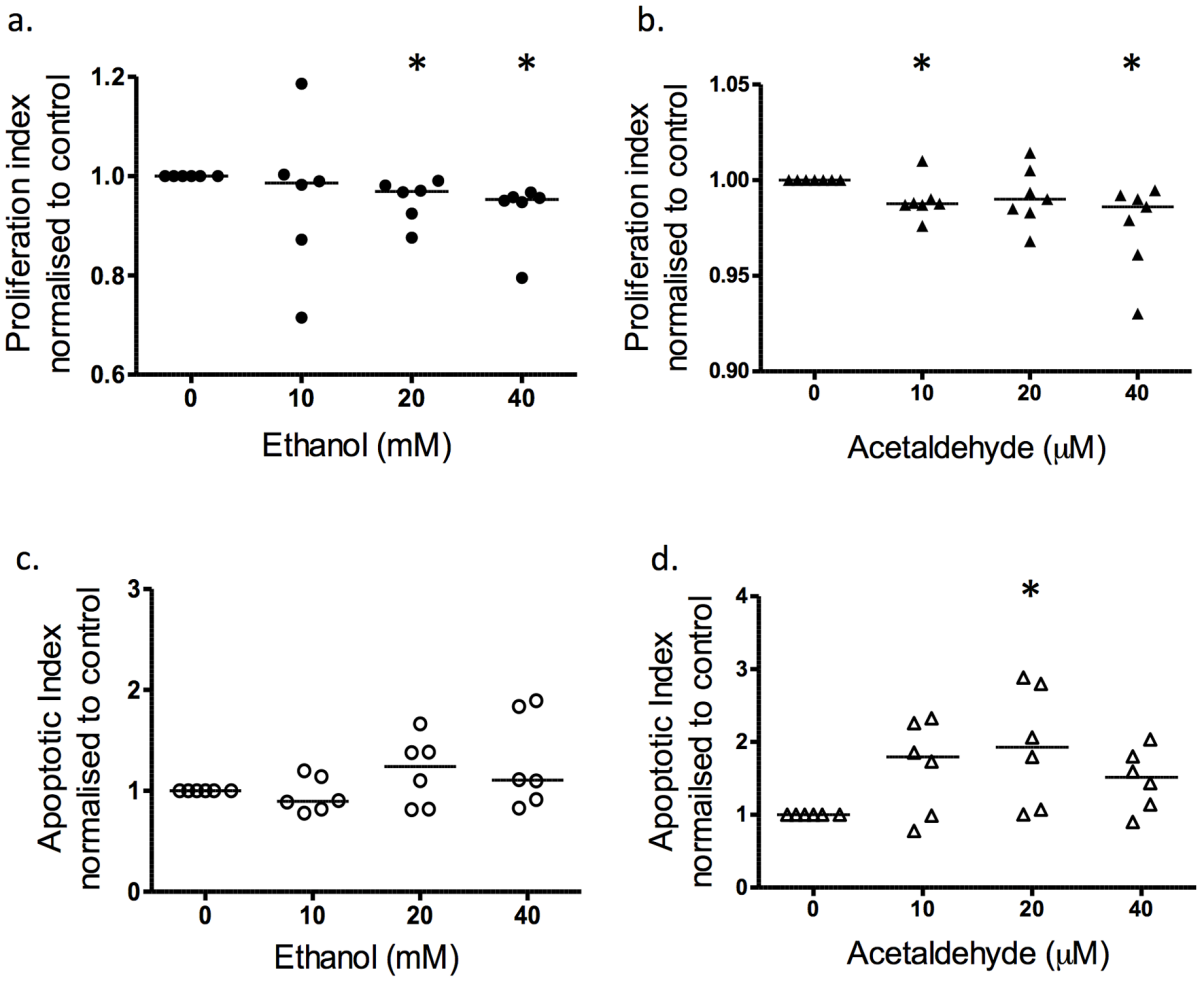
Effect of ethanol and acetaldehyde on the proliferation and apoptotic index of BeWo cells. The proliferation and apoptotic index as measured by the fraction of Ki67-positive cells or M30-positive cells, and normalised to control. (a) Ethanol exposure at 20 mM and 40 mM significantly reduced proliferation (p<0.05; N = 6); (b) Acetaldehyde treatment decreased the proliferation index at 10 µM and 40 µM (p<0.05; N = 7). Apoptotic M30-positive index of BeWo cells (c) was unaffected by ethanol exposure but (d) acetaldehyde increased apoptosis at 20 µM (p<0.05, N = 6). Statistical analysis with Wilcoxon signed-rank. *p<0.05.

Ethanol reduced the population of Ki67-positive (in-cycle) BeWo cells by 5% at 20 and 40 mM (Figure 2 a; p<0.05; N = 6). There was a decrease in Ki67-positive cells at 10 µM and 40 µM acetaldehyde (Figure 2b; p<0.05; n = 7). Apoptosis in BeWo cells was not affected by ethanol exposure (Figure 2 c; p>0.05; N = 6), however acetaldehyde treatment increased the apoptotic index at 20 µM (Figure 2d; p<0.05; N = 6).

**Figure 3:**
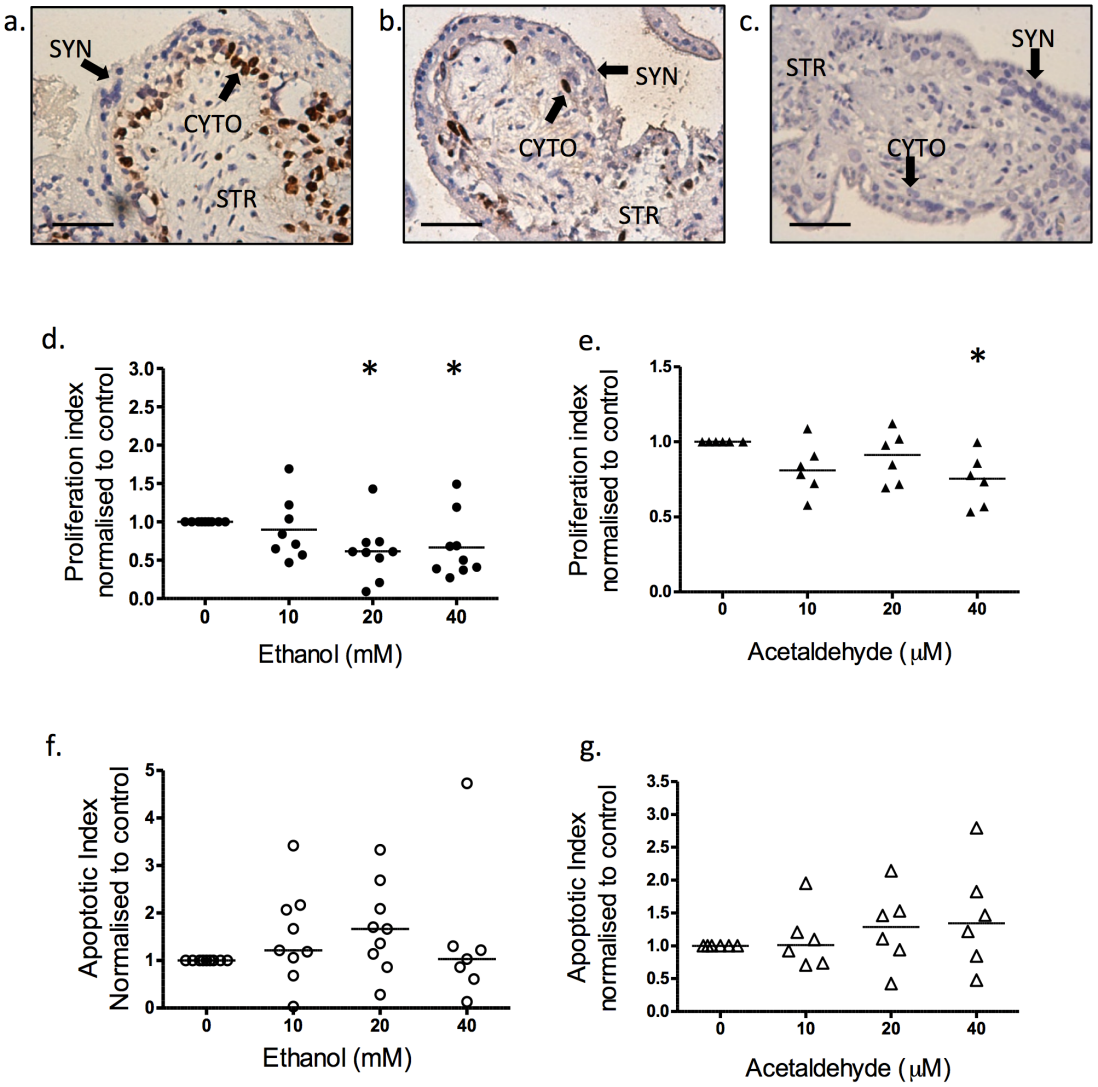
Effect of ethanol and acetaldehyde on the proliferation and apoptotic index of first trimester tissue. The proliferation and apoptotic index as measured by the fraction of Ki67-positive cells or M30-positive cells and normalised to control. (a) Immunostaining of Ki67 positive cytotrophoblasts in untreated first trimester tissue, (b) after treatment with 40 mM ethanol and (c) negative control with non-immune IgG. (d) Proliferation index of ethanol-exposed first trimester explants. At 20 mM and 40 mM, ethanol reduced cytotrophoblast proliferation (p<0.05; N = 9). (e) Proliferation index of acetaldehyde-exposed first trimester explants decreased proliferation at 40 µM only (p<0.05; N = 6). (f) Apoptotic index of explants after treatment with ethanol did not affect apoptosis (p>0.05; N = 7–9). (g) Treatment with increasing concentrations of acetaldehyde also did not affect apoptosis (p>0.05; N = 6). Statistical analysis with Wilcoxon signed-rank. *p<0.05. Scale bar = 50 micron.

In first trimester tissue, ethanol exposure at the middle to highest concentrations 20 mM and 40 mM, reduced Ki67-positive proliferative index by up to 40%, as compared to untreated control (Figure 3a, b and d; p<0.05; N = 9). Exposure to acetaldehyde at the highest concentration (40 µM) also reduced proliferation (Figure 2e; p = 0.03; N = 6). Exposure to ethanol did not increase apoptosis (figure 3f; p>0.05; N = 7–9), similar to acetaldehyde (Figure 3g; p>0.05; N = 6). The villous stroma showed no observable response to ethanol exposure at any of the concentrations used (Figure 3a and b; numerical data not shown, N = 6).

### Effect of Ethanol and Acetaldehyde on Placental Amino Acid Transport

**Figure 4:**
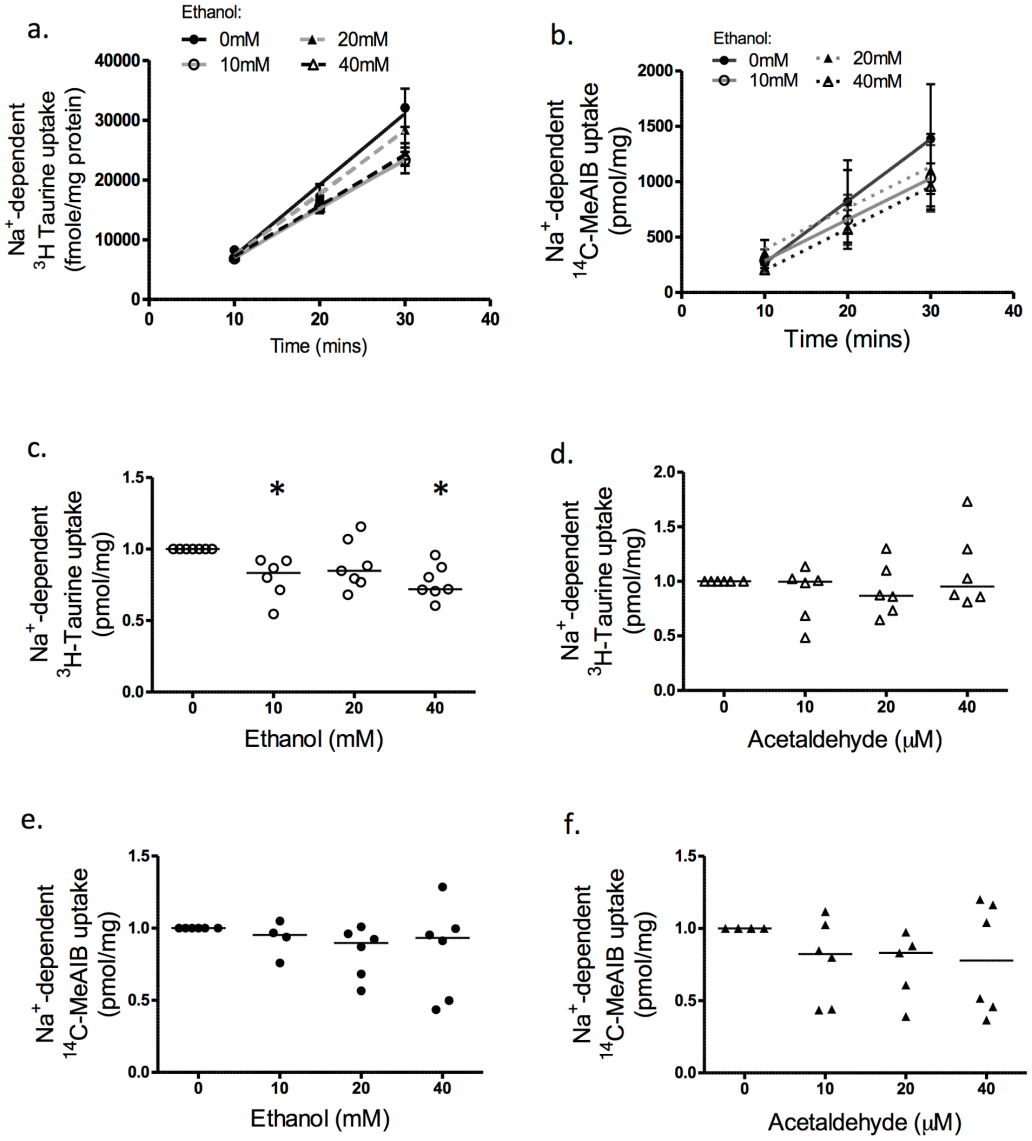
Effect of alcohol and acetaldehyde on system β and System A activity in BeWo cells. Na^+^-dependent ^3^H taurine uptake (system β activity) and Na^+^-dependent ^14^C-MeAIB uptake (system A activity) of BeWo cells over time. (a) system β activity and (b) system A activity, over 10, 20 and 30 minutes (Mean 23282±4809, SE = 2151, N = 6). The least squares linear regression shows that uptake was linearly related to time in all cases (p≤0.05). (c) System β activity of BeWo cells at 30 minutes. Ethanol treatment at 10 mM and 40 mM significantly decreased transporter activity (p<0.05; N = 6). (d) Acetaldehyde exposure did not change system β activity after 30 minutes (N = 6; p>0.05). (e) System A activity was not affected by ethanol or (f) acetaldehyde (N = 6; p>0.05). Statistical analysis with Wilcoxon signed-rank. *p<0.05 vs control.

Experiments were performed to determine the time course of uptake of ^3^H taurine and ^14^C-MeAIB into BeWo cells. Na^+^-dependent uptake was linear over 10–30 min for both radiolabelled amino acids and the regression line extrapolated close to the origin, indicating that transport was at the initial rate (Figure 4a and 4b). Therefore, the effects of treatments were compared to control at 30 minutes for both BeWo cells and first trimester explants.

Uptake of radiolabelled ^3^H taurine by BeWo cells was significantly reduced after exposure to ethanol at all the concentrations used (Figure 4c; p<0.05; N = 6). Surprisingly, exposure to acetaldehyde (N = 5) had no significant effect on taurine uptake at any of the concentrations used (Figure 4d). Furthermore there was no effect of ethanol or acetaldehyde exposure on ^14^C-MeAIB uptake at any concentration (Figure 4e and 4f; p>0.05; N = 5).

**Figure 5:**
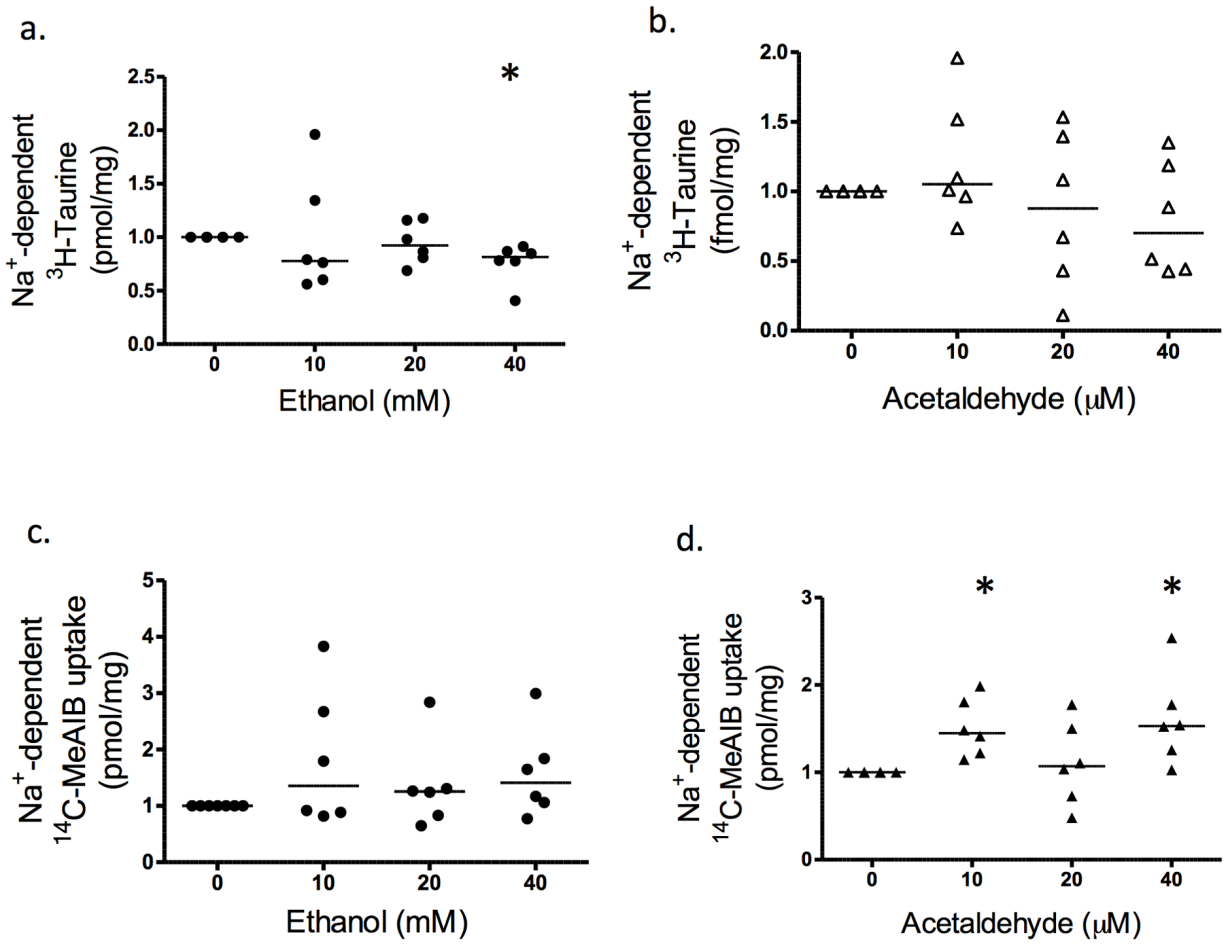
Effect of alcohol and acetaldehyde on system β and System A activity in first trimester placenta. Na^+^-dependent ^3^H taurine uptake (system β activity) and Na^+^-dependent ^14^C-MeAIB uptake (system A activity), of first trimester placental explants, measured at 30 min and normalised to control. (a) Ethanol at 40 mM significantly reduced system β activity of first trimester placental explants (p<0.05; N = 6) but (b) acetaldehyde was without effect. (c) Ethanol did not affect system A activity at any of the concentrations used, but (d) acetaldehyde at 10 µM and 40 µM significantly increased system A activity (p<0.05; N = 6). Statistical analysis with Wilcoxon signed-rank. *p<0.05 vs control.

In first trimester placental explants, ethanol inhibited system β taurine transporter activity at the highest concentration, 40 mM (figure 5a; p<0.05; N = 6). Acetaldehyde exposure did not affect placental explant system β activity at any of the concentrations used (Figure 5b). As for BeWo cells, ethanol did not significantly affect ^14^C-MeAIB uptake by placental explants (figure 5c, N = 5). However, acetaldehyde increased uptake at 10 µM and 40 µM (figure 5d; p<0.05).

## Discussion

The first trimester of pregnancy is crucial for placental development, which in turn provides for organogenesis and fetal growth [41]. To set clinically relevant experimental concentrations, we examined the literature on circulating alcohol concentrations that might be achieved during binge drinking. A blood alcohol concentration of 0.08% by volume (approximately 17.7 mM) is the defined intoxication limit for driving in the UK and USA. Literature on peak blood-alcohol suggests that 40 mM causes intoxication in a normal population; 40 mM alcohol can result from an exposure equivalent to 4–5 units (3–4 standard drinks). The average peak blood acetaldehyde concentration is in the range 26–43 µM [12], [15], [16], [34], [42], [43]. Pharmacological studies in animals have used as much as 50–100 mM ethanol administered daily [19]. We have shown that ethanol or acetaldehyde at clinically relevant concentrations (≤40 mM and ≤40 µM respectively) has adverse effects on two key aspects of trophoblast function: proliferation and nutrient transport. These placental effects suggest potential mechanisms by which maternal alcohol consumption could impact on fetal development.

Placental insufficiency, diagnosed at term, has been documented as a leading cause of FGR [11], [44], and growth restriction is associated with extreme chronic level alcohol consumption [19], [23], [24]. Although genetic differences in alcohol metabolism generates conflicting data in human pregnancies, placentas obtained from women who have consumed alcohol during pregnancy contain more villous infarction, thrombosis and vascular abnormalities, compared to non-exposed pregnancies [45]–[47]. Furthermore, trophoblast proliferation is reduced in FGR [48].

Primary cultured cytotrophoblasts rapidly exit the cell cycle, but we have shown that proliferation can be studied in placental explant cultures which retain the naturally occurring polarity and intrinsic environment of the trophoblast epithelium [49]–[52]. Our results indicate that ethanol above 20 mM and acetaldehyde at 40 µM attenuated cytotrophoblast but not stromal cell proliferation in first trimester placental tissue. Term human trophoblasts exposed to ethanol have also been previously shown to decrease in proliferation, but in concentrations up to 100 mM [34], [53]. The resulting changes in placental development are reflected by significant reduction in fetal weights [19], [24]. These data suggest that alcohol exposure as well as acetaldehyde can adversely affect placental growth throughout pregnancy.

SGHPL-4 cells were derived from transformed first trimester extravillous cytotrophoblasts and have retained invasive characteristics [49], [54]. A strong trend towards inhibition of migration was observed through a matrigel barrier after ethanol and acetaldehyde exposure (ethanol at 40 mM and acetaldehyde at 20 µM and 40 µM p = 0.055–0.06); limitations of access to cells for this study precluded more experiments to confirm a statistically significant effect. However, other studies using immortalised extravillous cytotrophoblast cells have shown that motility can be inhibited at approximately 17 mM of alcohol over 48 hours [55]. In rat, ethanol exposure reduces the number of invasive trophoblast giant cells [56] and induces cell death in the spongiotrophoblast layer [19]. Proficient extravillous cytotrophoblast invasion in the early first trimester plays a critical role in transforming maternal arteries, supporting a stable low pressure supply of nutrients and gases [57]. Defective invasion of extravillous cytotrophoblasts with reduced arterial remodelling is associated with a range of pregnancy pathologies [9], [10] including FGR [58]. Further work will be needed to assess the impact of alcohol consumption on invasion and vascular remodelling in the first trimester placental bed.

The placental system β amino acid transporter is responsible for providing the fetus with taurine, a sulphur-amino acid with anti-oxidant properties that may be neuroprotective during fetal development [37]. Maternal taurine deficiency is also associated with arrest in fetal growth [59], [60], affecting beta cell development and insulin sensitivity [61]. This study demonstrates detrimental effects of clinically relevant ethanol concentrations on the taurine transporter in both BeWo, an epithelioid trophoblast cell line of lower invasive potential [38], [62]–[64], and explants of first trimester placenta. Even at the lowest concentration used, equivalent to an approximate a blood alcohol concentration of 0.05 (% of volume), is able to disrupt normal taurine transporter function. In humans, taurine is maternally derived during pregnancy as fetal production is insufficient to support development [60]. Maternal taurine deprivation in animal models demonstrates decreased fertility and increased incidence of fetal resorption and stillbirth in feline and rodent models [59], [65]–[67]. Surviving neonates have lower birth weight, decreased brain weight and experience slower growth [59], [60]. In the complete absence of taurine transporters, knock-out mice are significantly growth restricted at birth, and have visual, auditory, and muscular alterations in adulthood compared to their wild types [68].

Reduced placental taurine concentrations have been correlated with FGR [69], where system β activity is significantly restricted in placentas of FGR fetuses compared to normal pregnancies [70], [71]. This reduction in placental system β activity is observable even in pregnancies with higher risk of FGR [72]. At 40 mM alcohol, the inhibition of taurine transporter function is potentially exposing the fetus to periods of significant deprivation [73]. Thus taurine deprivation, secondary to ethanol exposure, may contribute towards the varying degrees of neurological and behavioural differences and growth restriction seen in FASD affected children.

In addition, intracellular taurine behaves as an osmolyte, regulating the osmotic balance during cellular hydration, which plays an essential role in balancing proliferation and apoptosis [74], [75]. The role of taurine is likely to be important in placental as well as fetal development as siRNA knockdown of system β inhibits differentiation of placental trophoblasts in vitro [76]. Addition of taurine in vitro has also shown to increase rat neural stem cell proliferation and secretion of synapse developmental proteins [77]. When utilised as a dietary supplement after ethanol exposure in animal models, taurine markedly reduces the rate of abnormal neuronal migration and decreases the number of brain lesions [78]. Further work is required to investigate whether supplementation can improve outcomes where high levels of alcohol exposure are known to have occurred.

Acetaldehyde, on the other hand, did not affect taurine transporter activity. Acetaldehyde has been suggested to enhance toxicity of ethanol and be the more toxic teratogen [27], [30]. Other studies have shown acetaldehyde to have a detrimental effect on α-amino isobutyric acid uptake (which is transported via system A) in both human and sheep models, but at concentrations over a 1000 fold more than the concentrations used in this study [16], [26]. These pharmacological concentrations of acetaldehyde would not be found in a biological system as the metabolism of acetaldehyde plateaus at 26.5 µM for an average population and 42.3 µM for chronic alcoholics [42], [79].

The system A amino acid transporter is a sodium-dependent transport system of small neutral amino acids that has shown to be associated with FGR, a key feature of FASD [6]. In contrast to the taurine transporter, no effects of ethanol on the activity of system A amino acid transport in either BeWo cells or in first trimester placental explants were observed, in agreement with previous work, with in vitro models, using placental explants exposed to 60 mM ethanol [26], [80]. Acetaldehyde, applied at concentrations much lower than in other studies [28], [66], [81] induced a small increase in ^14^C-MeAIB uptake in placental explants, but not in BeWo cells. This observation may reflect a compensatory increase in response to an adverse environment as seen in other physiological systems when exposed to a chemical stimulus, as first trimester explants retain their phenotype in culture [82]. There is a potential capacity for functional compensation in first trimester tissue, but not in cells, because of the diversity of cell types. As a trophoblast cell layer model, BeWo cells are commonly used to assess membrane transport as they express similar functional transport receptors [83]–[87] and retain the trophoblastic property of fusion into a multinucleated layer [88]. Detrimental effects have been described at pharmacological concentrations of acetaldehyde (x20 to 2000 greater than those found biologically) on system A transport of the amino acid, valine, in rat placental explants [89] and on the neutral system A transport of lithium-dependant L-alanine in human term explants [79]. This indicates that acetaldehyde may have selective effects on different amino acid transporters; however, though toxic at pharmacological concentrations, it does not affect MeAIB transport at clinically relevant levels.

Alcohol consumption is common in many cultures [90] and alcohol over-use by young women is an increasing public health issue [1]. This study has shown that short, acute exposure to ethanol, at levels readily achieved in a single sitting, negatively affects first trimester placental cell growth, trophoblast migration and the function of an amino acid transporter vital for normal neurological development. This implicates adverse developmental effects of ethanol at the earliest stages of placental development, with potentially major developmental effects for the fetus, and suggests that abstinence in the early stages of pregnancy is the safest clinical advice.
